# What Patients Find on the Internet When Looking for Information About Percutaneous Coronary Intervention: Multilanguage Cross-sectional Assessment

**DOI:** 10.2196/41219

**Published:** 2022-12-06

**Authors:** Cristina M Șulea, Valentin Nădășan, Tatiana Ursachi, Paul-Cătălin Toboltoc, Theodora Benedek

**Affiliations:** 1 George Emil Palade University of Medicine, Pharmacy, Science, and Technology of Targu Mures Targu Mures Romania; 2 Lucian Blaga University of Sibiu Sibiu Romania

**Keywords:** percutaneous coronary intervention, consumer health informatics, internet, health education, health information, quality, reliability, informed decision-making, credibility, content quality, medical information

## Abstract

**Background:**

The internet provides general users with wide access to medical information. However, regulating and controlling the quality and reliability of the considerable volume of available data is challenging, thus generating concerns about the consequences of inaccurate health care–related documentation. Several tools have been proposed to increase the transparency and overall trustworthiness of medical information present on the web.

**Objective:**

We aimed to analyze and compare the quality and reliability of information about percutaneous coronary intervention on English, German, Hungarian, Romanian, and Russian language websites.

**Methods:**

Following a rigorous protocol, 125 websites were selected, 25 for each language sub-sample. The websites were assessed concerning their general characteristics, compliance with a set of eEurope 2002 credibility criteria, and quality of the informational content (namely completeness and accuracy), based on a topic-specific benchmark. Completeness and accuracy were graded independently by 2 evaluators. Scores were reported on a scale from 0 to 10. The 5 language subsamples were compared regarding credibility, completeness, and accuracy. Correlations between credibility scores on the one hand, and completeness and accuracy scores, on the other hand, were tested within each language subsample.

**Results:**

The websites’ compliance with credibility criteria was average at best with scores between 3.0 and 6.0. In terms of completeness and accuracy, the website subsets qualified as poor or average, with scores ranging from 2.4 to 4.6 and 3.6 to 5.3, respectively. English language websites scored significantly higher in all 3 aspects, followed by German and Hungarian language websites. Only German language websites showed a significant correlation between credibility and information quality.

**Conclusions:**

The quality of websites in English, German, Hungarian, Romanian, and Russian languages about percutaneous coronary intervention was rather inadequate and may raise concerns regarding their impact on informed decision-making. Using credibility criteria as indicators of information quality may not be warranted, as credibility scores were only exceptionally correlated with content quality. The study brings valuable descriptive data on the quality of web-based information regarding percutaneous coronary intervention in multiple languages and raises awareness about the need for responsible use of health-related web resources.

## Introduction

On account of its accessibility and interactivity, the internet has become a popular and widely used tool for independent medical documentation among the general public. The proportion of people who turn to the web-based environment in search of health-related information has been steadily increasing [1]. This practice, although regarded as convenient from the consumers’ point of view, has raised concerns among physicians, as the quality of the web-based medical information and the patients’ or caregivers’ ability to select relevant information are often seen as questionable [2]. Therefore, the negligent use of the internet may impact the physician-patient relationship and consumers’ medical decision-making, leading to unjustified fears (also known as ‘cyberchondria’), defiance of medical advice, or inclination toward self-diagnosis and self-treatment [3-6].

The interventional treatment of coronary artery disease is one of the topics of high interest among patients and caregivers, as the condition is one of the leading causes of morbidity and mortality globally [7]. The quality and reliability of the information available online on the subject of percutaneous coronary intervention (PCI) may have a considerable impact on the general public’s understanding of the technique, compliance, and outcomes of their therapeutic decisions. Hence, this study aimed to assess and compare the quality of information about PCI on a sample of English, German, Hungarian, Romanian, and Russian websites and to evaluate the reliability of credibility criteria as indicators of information quality. These aspects are hoped to provide valuable insight into the quality of web-based health information, not only on behalf of internet users and medical practitioners but also website owners and policy makers, to serve as foundation for the effective education of the general population on the topic of internet health-related documentation.

## Methods

### Sample Selection

**Figure 1:**
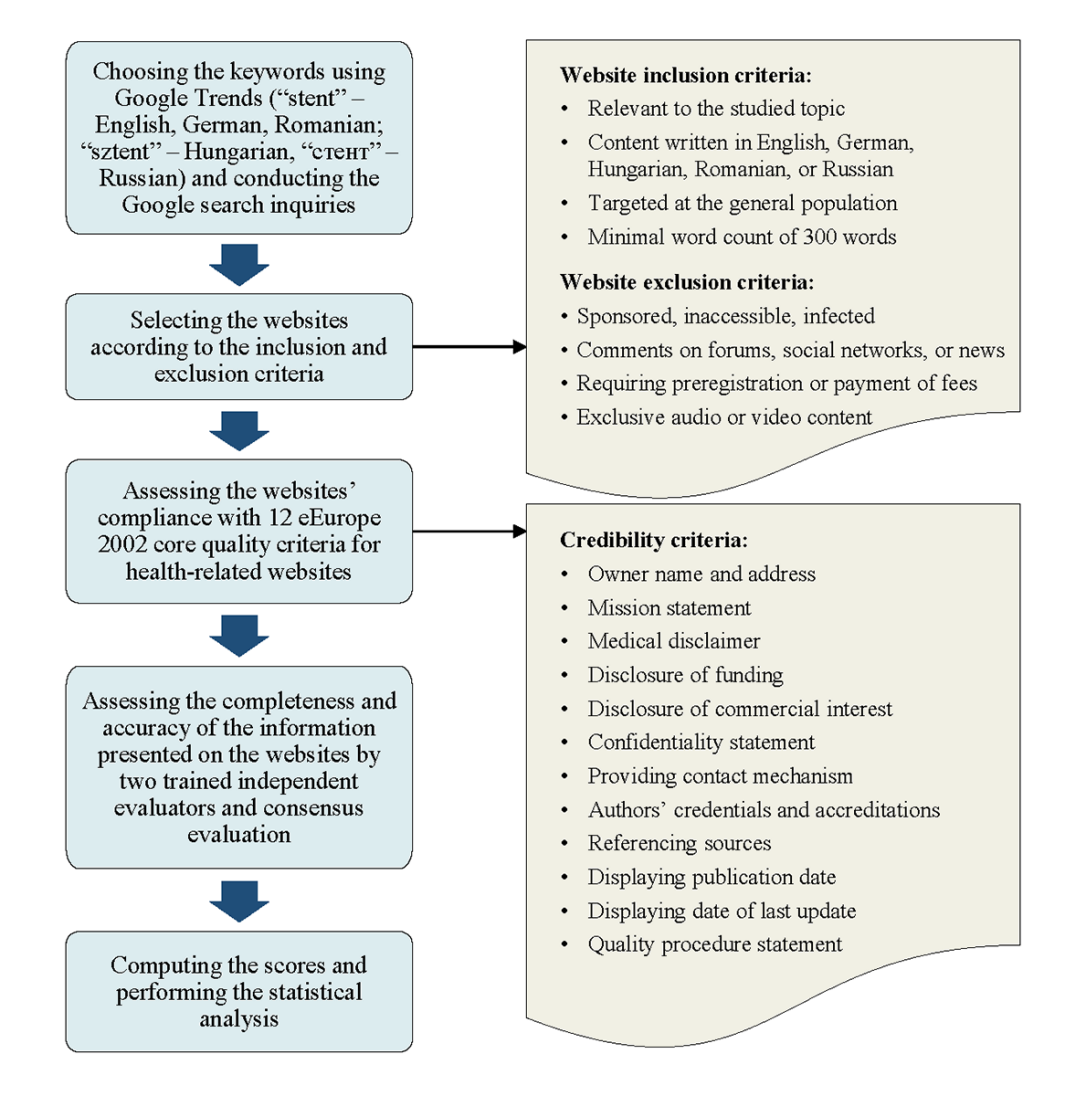
Flowchart representing the main steps of the study.

The research was designed as an observational cross-sectional study. Its sample consisted of 125 PCI-related websites intended for the general population in 5 languages (25 for each included language)—English, German, Hungarian, Romanian, and Russian. The Google Search engine was used to identify eligible websites, using “stent” as query term (used as such in English, German, and Romanian; “sztent” in Hungarian; and “стент” in Russian). The query terms were selected based on their popularity as shown by Google Trends, a tool that analyzes the frequency of top search queries in Google Search across various regions and languages. The links returned by the Google Search engine were screened according to a set of preestablished inclusion and exclusion criteria. To be included, a website had to address the subject of coronary stenting, presenting the information in the desired language and a minimum of 300 words. The information had to be targeted at internet users without medical education. Pages addressing subjects other than PCI, sponsored pages appearing in the top hierarchical positions in the results list, and infected or inaccessible pages were excluded. Websites consisting exclusively of audio or video content and websites allowing access only after registration or payment of a fee were also excluded. Similarly, web pages that presented the topic of interest in the form of news or comments on forums and social networks—in other words, pieces of information not meant to thoroughly present the subject of PCI—were not included in the sample. Websites deemed fit for inclusion were consecutively analyzed following a rigorous protocol, briefly illustrated in Figure 1.

The Google searches were performed in April 2019 for English, Hungarian, and Romanian language websites, while the Russian language inquiry took place in June 2019. German language websites were subsequently added to the study in November 2020. A total number of 83, 39, 121, 167, and 94 websites in English, German, Hungarian, Romanian, and Russian language, respectively, were screened until the acquisition of the 25 eligible links for each of the subsamples.

### Data Collection

Firstly, the examination was aimed at the websites’ general characteristics and their compliance with 12 general credibility criteria derived from the eEurope 2002 core quality criteria for health-related websites supported by the Commission of the European Communities [8] (Figure 1). Next, the selected pages underwent an exhaustive evaluation of their informational content based on a topic-specific benchmark (Multimedia Appendix 1) [9]. The benchmark was developed using published literature and evidence-based guidelines on the subject of interest as sources of information, in such a way that it covered the topic of PCI to an extent considered sufficient and comprehensible for nonprofessionals. It included information on the following aspects: definitions and introductory notions about PCI, types of coronary stents, indications for the procedure, preprocedural preparation of the patient, description of the procedure, the postprocedural period, what to know or do at home, risks, benefits, costs, other treatment options, general prevention and prophylaxis methods, as well as general warnings regarding alternative treatments. To ensure a practical grading manner, the benchmark was divided logically into 50 items. Their presence on the studied websites was evaluated regarding completeness (ie, the presence of the item on the studied website, evaluated in a binary fashion) and accuracy (ie, the extent to which the item was correctly presented on the studied website, graded on a 3-point scale). The benchmark was reviewed by medical professionals, specialists in the fields of cardiology and interventional radiology, from both Romania and the United States.

The data on the websites’ general characteristics and compliance with credibility criteria were collected by one operator, while the assessments regarding the websites’ informational content were performed independently by 2 evaluators for all websites. The websites’ compliance with the 12 selected eEurope 2002 credibility criteria was assessed in a binary fashion, with 1 point given to every criterion that was met. Based on the obtained sum, the relative credibility score of the given web page was calculated as previously described by Nădăşan et al [9]. Similarly, based on the points awarded for completeness and accuracy for each website, their relative completeness and accuracy scores were computed. All relative scores were reported on a 0-10 scale. The resulting scores were categorized as very poor (0-2), poor (2.1-4), average (4.1-6), good (6.1-8), or very good (8.1-10). The analyzed data are available in Multimedia Appendix 2.

### Statistical Analysis

For each included website, the degree of agreement between the 2 evaluators was assessed using the Cohen kappa statistic, a test that measures interrater reliability and is regarded as more robust than simply computing the percentage of agreement, as it adjusts for agreement occurring by chance. Kappa coefficients may range from −1 to 1. A kappa value of 1 indicates perfect agreement, while a value of 0 corresponds to the rate of agreement expected by chance alone. In our study, a coefficient of less than 0.8 prompted a reevaluation to reach a consensus. The Kolmogorov-Smirnov test was used to analyze the normality of the data, based on which the comparisons of data with normal and nonnormal distributions were performed using the 2-tailed Student *t* test and the 2-tailed Mann-Whitney test, respectively. The correlations between credibility scores on the one hand and completeness and accuracy scores on the other hand were analyzed using the Spearman rank correlation test.

The statistical analyses were conducted using IBM SPSS Statistics for Windows (version 22.0; IBM Corp). The threshold value for statistical significance was set at a value of α=.05. The obtained scores are presented as mean (SD).

## Results

Of the 125 included websites, nearly two-thirds had a general medical approach, comprising information belonging to multiple medical specialties. Most of the web pages were owned by private or state medical service providers. In terms of purpose, the pages were predominantly educational. As far as their format was concerned, the most often identified were company presentation pages. Most websites were characterized by a conventional medicine approach. The detailed distribution of the studied websites according to their general characteristics is shown in Table 1.

**Table 1:** The absolute (n) and relative (%) frequencies of the websites based on their general characteristics.

General characteristics		Values, n (%)
**Specialization**		
	Single medical specialty	40 (32)
	Multiple medical specialties	85 (68)
**Website ownership**		
	Foundation or nongovernmental organization	15 (12)
	Private or state health care provider	43 (34.4)
	Commercial company	23 (18.4)
	Manufacturer or distributor of medical supplies and equipment	6 (4.8)
	Private person	3 (2.4)
	Educational or research institution	9 (7.2)
	Unidentifiable	26 (20.8)
**Main purpose**		
	Educational	68 (54.4)
	Commercial	48 (38.4)
	Socialization or support	9 (7.2)
**Website format**		
	Thematic	8 (6.4)
	Medical or general portal	37 (29.6)
	Electronic publication	21 (16.8)
	Company presentation page or web-based shop	52 (41.6)
	Blog or personal page	3 (2.4)
	Other	4 (3.2)
**Medical paradigm**		
	Conventional medicine	108 (86.4)
	Mixed (ie, alternative and conventional) approach	5 (4)
	Unidentifiable	12 (9.6)

**Figure 2:**
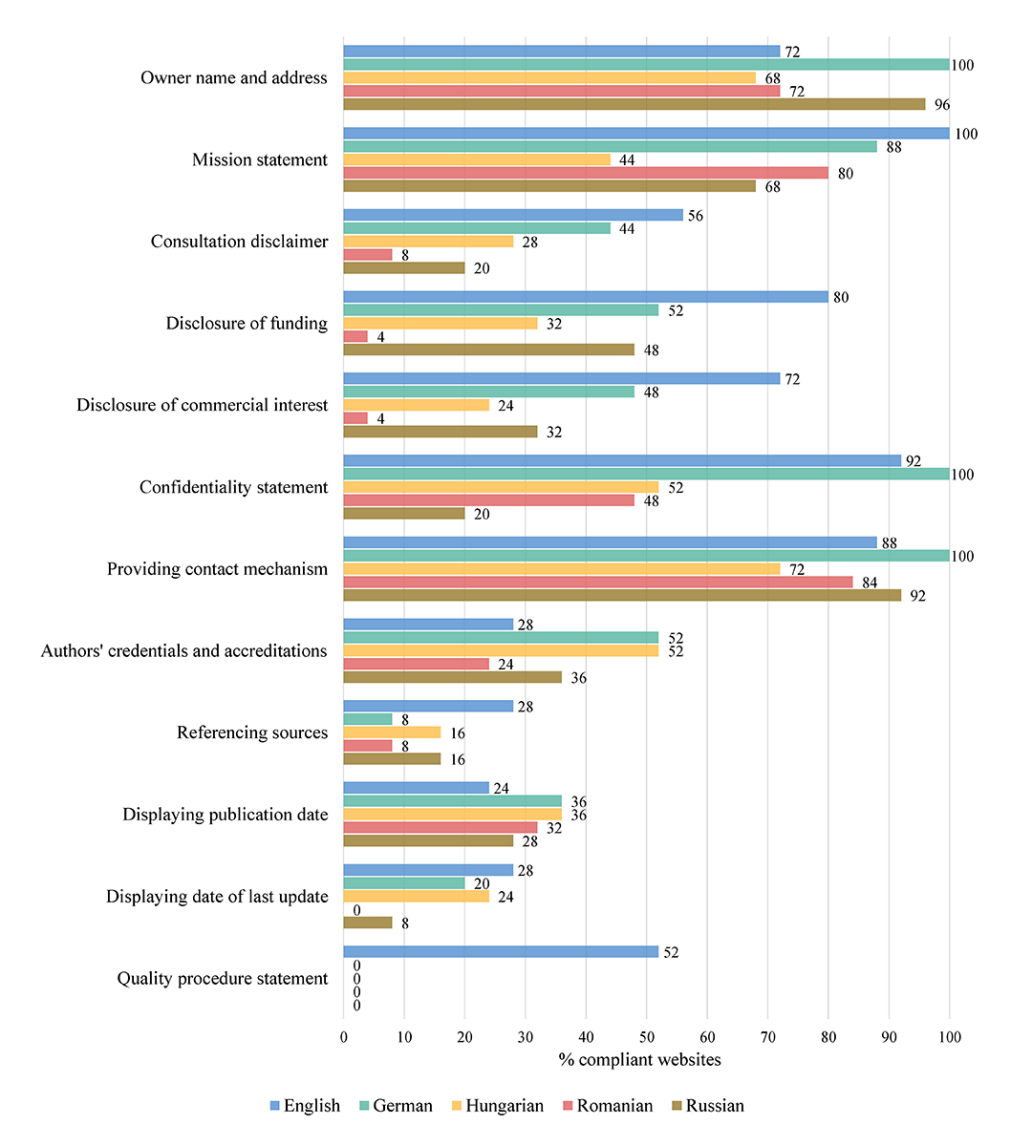
The websites’ level of compliance with the credibility criteria by language subsample.

The websites’ overall compliance with the selected eEurope 2002 credibility criteria was highly variable, with some criteria being fulfilled to a greater extent (providing a direct contact mechanism: 87.2%; including the owner’s name and address: 81.6%; and providing a mission statement: 76%), while others were identified on few of the included web pages (providing a quality procedure statement: 10.4%; including referencing sources: 15.2%; and displaying the date of last update: 16%). The remaining credibility criteria were identified on approximately one to two-thirds of the studied pages (displaying the publication date of the articles and a consultation disclaimer: 31.2% each; including disclosure of commercial interest: 36%; including the authors’ credentials and accreditations: 38.4%; providing a declaration of funding: 43.2%; and offering a confidentiality statement: 62.4%). Figure 2 illustrates the compliance of each of the 5 groups of websites with the selected credibility criteria.

**Figure 3:**
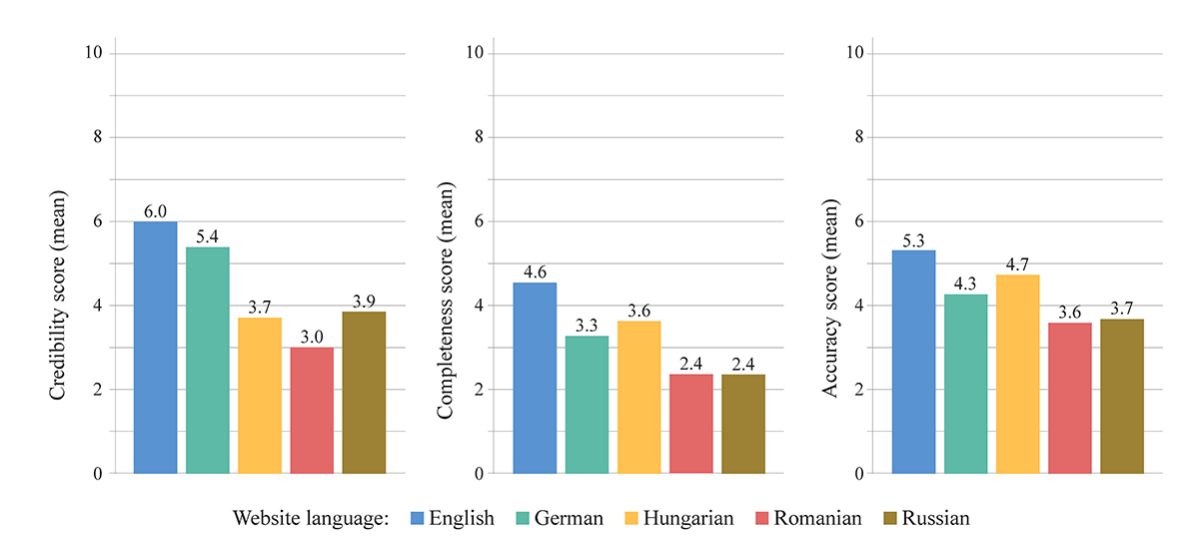
The websites’ relative credibility, completeness, and accuracy scores by language subsample.

The overall mean relative credibility, completeness, and accuracy scores were 4.4 (SD 2.2), 3.2 (SD 1.6), and 4.3 (SD 1.6), respectively. The mean scores of the 5 language subsamples are summarized in Figure 3. The results of the comparison and correlation tests are presented in Table 2 and Table 3, respectively.

**Table 2:** The results of the comparison tests between language subsamples regarding the relative credibility score (RQS), relative completeness score (RCS), and relative accuracy score (RAS). *P* values with statistical significance are emphasized in italics.

Compared language subsamples	*P* values		
	RQS	RCS	RAS
English vs German	.19^a^	*.002* ^a^	*.004* ^a^
English vs Hungarian	*.001* ^a^	*.04* ^a^	.11^a^
English vs Romanian	*<.001* ^b^	*<.001* ^b^	*<.001* ^a^
English vs Russian	*<.001* ^b^	*<.001* ^a^	*.001* ^a^
German vs Hungarian	*.01* ^a^	.33^a^	.18^a^
German vs Romanian	*<.001* ^b^	*.01* ^b^	.06^a^
German vs Russian	*<.001* ^b^	*.01* ^a^	.18^a^
Hungarian vs Romanian	.37^b^	*.002* ^b^	*.004* ^a^
Hungarian vs Russian	.94^b^	*.002* ^a^	*.02* ^a^
Romanian vs Russian	.16^b^	.94^b^	.88^a^

^a^Student *t* test.

^b^Mann-Whitney test.

**Table 3:** The results of the Spearman rank correlation tests between the credibility scores and informational content quality scores for all the language subsamples (*P* values with statistical significance are emphasized in italics).

Language subsamples	Tested variables	Correlation coefficient	*P* values
**English**			
	Credibility vs completeness	0.3439	.09
	Credibility vs accuracy	0.0578	.78
**German**			
	Credibility vs completeness	0.4672	*.01*
	Credibility vs accuracy	0.3994	*.04*
**Hungarian**			
	Credibility vs completeness	–0.3507	.08
	Credibility vs accuracy	–0.1940	.35
**Romanian**			
	Credibility vs completeness	–0.2592	.21
	Credibility vs accuracy	–0.3169	.12
**Russian**			
	Credibility vs completeness	0.1280	.54
	Credibility vs accuracy	–0.0645	.75

## Discussion

### Principal Findings

As far as the websites’ credibility is concerned, the obtained scores were average at best, with English and German language websites acquiring the highest results (mean 6.0, SD 1.8 and mean 5.4, SD 1.3, respectively), significantly higher than those of Hungarian, Romanian, and Russian language websites, which were graded as poor. Although certain criteria (eg, displaying the owner’s name and contact information as well as providing a direct contact modality) were largely met by web pages in all 5 languages, other criteria (eg, providing authors’ credentials and accreditations, bibliographic references, articles’ dates of publication and last update, or offering a quality procedure statement) were scarcely included. This may raise a red flag since authorship and providing references are perceived as important indicators of medical information reliability. Apparently, Hungarian, Romanian, and Russian language websites’ owners may not pay enough attention to credibility or are unaware of this aspect. These findings are consistent with previously published literature investigating the credibility of web-based information about different medical topics [10-12]. Moreover, compliance with the credibility criteria for health-related websites as measured by the Health on the Net Code of Conduct has been shown to vary largely depending on the type of organization and health conditions [13].

Regarding the evaluation of the websites’ informational content, the completeness and accuracy of the data about PCI in the 5 studied languages were found to be rather unsatisfactory, with the obtained scores only getting average and poor labels. In terms of completeness, English language websites acquired the highest scores (mean 4.6, SD 1.6), significantly higher than those of the websites in the other 4 language subsamples. German and Hungarian language websites also performed significantly better than the Romanian and Russian websites. In terms of accuracy of data, English language websites had significantly higher scores than German, Romanian, and Russian but not Hungarian websites, which had significantly higher accuracy scores than Romanian and Russian websites. A relative superiority of English language health-related websites compared to Spanish ones has been observed as early as 2001 in a study covering multiple medical conditions (ie, breast cancer, depression, obesity, and childhood asthma), and it was more recently compared to Turkish websites focusing on an orthopaedic intervention [14,15]. Leaving aside methodological differences, the results of these studies call attention to possible language-mediated inequities and suggest that a multilingual approach to web-based documentation may provide more complete coverage of the topic. Apparently, in some countries such as Romania, the low quality of web-based information about PCI seems to be in line with the low number of PCIs per million individuals, as shown by the latest statistics published by the European Society of Cardiology [16]. Efforts to increase the quality of web-based information about PCI would be a reasonable step in countries where access to these interventions is wanting.

The correlation assessments did not find statistically significant relationships between the credibility of the PCI-related websites and the quality of their informational content, with one exception. In the case of German language websites, the compliance with credibility criteria exhibited statistically significant, moderate strength correlations with the websites’ coverage of the topic (ie, completeness and accuracy). The lack of consistent correlations between credibility and content quality has been previously reported in investigations focusing on various medical conditions, such as stroke and depression or procedures such as first aid instructions in case of choking [17-19]. The results may suggest that the selected credibility criteria are not reliable indicators of information quality on PCI-related websites in the studied languages, and therefore, cannot be recommended to nonprofessionals as marks of trustworthiness.

### Inferences

Despite the growing demand for web-based medical information, the recognition of the importance of patient participation in medical decision-making, and the impact of health-related web content on consumer health [20,21], the credibility and quality of websites about PCI—a procedure globally used to mitigate the consequences of the most common type of heart disease—has not yet been rigorously analyzed. To the best of our knowledge, this is the first study analyzing and comparing the quality of information about PCI on English, German, Hungarian, Romanian, and Russian websites aimed at the general population. The results of this study may be used to raise awareness among internet users about the limitations and potential hazards of using the web as a source of information about PCI. Engaging in safe internet browsing is crucial, as it may prevent poor decision-making and potential complications caused by delayed intervention as well as a deterioration of the physician-patient relationship [20,22]. Although the web-based environment is easily accessible and convenient, it is highly advisable that consumers engage in web-based medical documentation with precaution and always turn to medical professionals for advice. Furthermore, medical practitioners should fully acknowledge the reality of e-patients and handle it appropriately [23,24]. In this regard, the involvement of health professionals in the development of plain language and accurate web-based health resources and their involvement in providing guidance to patients with inadequate health literacy in accessing proper information on the internet could prove beneficial [25].

### Strengths and Limitations

It is worth noting the strengths of this study. First, the inclusion of multiple languages, of which at least three are spoken by vast numbers of individuals worldwide, allows for the extrapolation of the results and recommendations to a large population. For instance, according to Ethnologue [26], English is the most widely used language around the globe, being spoken by approximately 1.5 billion people across more than 140 countries. Moreover, both Russian and German are among the top 15 most widely spoken languages, with nearly 260 and 135 million speakers, respectively.

Second, most of the previously published studies focus on assessing health-related web-based sources based on credibility (reliability), readability, or design criteria (eg, the Health on the Net Code, JAMA score, DISCERN score, Flesch-Kincaid readability test, and SMOG Readability Index) [27]. As acknowledged by the authors of the DISCERN instrument [28], not even this tool was designed to actually measure the scientific fidelity of the information. Our study addresses not only the credibility or reliability dimension but also the quality of the content by evaluating the completeness and accuracy of information based on an evidence-based, topic-specific quality benchmark.

Third, to minimize subjectivity and the human error factor, the content quality assessments were conducted by 2 independent evaluators.

The main limitations of the study are related to some inherent traits of web-based research. Internet users may turn to various search engines or use different keywords, consecutively obtaining different search results [29]. Moreover, the continuously changing dynamics of the web-based environment make it virtually impossible for the results of this study to be precisely replicated. Additionally, the sample size may be argued as small. However, most internet users limit their inquiries to the first Google Search results page (on average, the first 10 search results) [30]. Therefore, by simulating a popular search strategy among lay internet users, we are confident that our results are likely to reflect common experiences. The study brings valuable descriptive data on the quality of web-based PCI-related information in multiple languages and has the potential to raise awareness about the need for responsible use of health-related web resources.

### Conclusions

The quality and reliability of the web-based information about PCI on English, German, Hungarian, Romanian, and Russian websites are rather unsatisfactory, and there are significant differences in the quality of information across the studied languages. It is safe to say that the internet does not provide the general public with good-quality medical information on the aforementioned topic. Moreover, the selected credibility criteria cannot be recommended as consistent indicators of information quality. Further efforts ought to be made by website developers to improve the trustworthiness of web-based health information. Since medical websites have become one of the most trusted sources of health-related documentation, it is crucial to both parties involved in the medical act (ie, lay users and medical practitioners) to develop awareness of the potential dangers internet documentation may pose. Our results could contribute to advances in the fields of preventive medicine or public health, supporting the importance of internet education among the general population.

## Appendix

Multimedia Appendix 1 Topic-specific benchmark for assessing the quality of information about percutaneous coronary intervention.

Multimedia Appendix 2 Quality assessment data and the results of statistical analyses.

## Abbreviations

PCI: percutaneous coronary intervention
