# Supplementary material for: What Patients Find on the Internet When Looking for Information About Percutaneous Coronary Intervention: Multilanguage Cross-sectional Assessment
Source: J Med Internet Res. 2022 Dec 6;24(12):e41219. doi: 10.2196/41219 (PMC9768670; doi:10.2196/41219)
Supplement: Multimedia Appendix 1 [file jmir_v24i12e41219_app1.pdf]

Benchmark – Percutaneous Coronary Intervention (PCI)

**1. Definitions, introduction, general information**	**1.** A stent is a small, expandable mesh tube that is placed into a narrowed (blocked) artery as a part of a procedure called percutaneous coronary intervention (PCI), also known as coronary angioplasty. The stent not only helps enlarge a segment of the artery to improve blood flow, but also prevents the vessel from narrowing or closing again.<br>**2.** Arteries are blood vessels that carry oxygenated blood from the heart towards other parts of the body. The coronary arteries are the vessels that provide the cardiac muscle with oxygen and nutrients needed for its normal function. When a blockage of the coronary arteries occurs, depending on its severity, it may cause chest pain (angina pectoris) or even a heart attack.<br>**3.** The most frequent cause of arterial narrowing (stenosis) is atherosclerosis, which consists of plaque build-up on the internal surface of the blood vessel, due to various risk-factors, some of which are nonmodifiable (family history, advanced age, genetic abnormalities, male gender) and others which depend on the individual lifestyle and can be modified or prevented (high cholesterol levels, smoking, sedentary lifestyle, unhealthy diet, obesity, high blood pressure, diabetes).
**2. Types of coronary stents**	The main types of stents available at the moment are:<br>**4.** Bare-metal stents (BMS) = mesh-like tube without a covering or coating, made of stainless steel or different alloys;<br>**5.** Drug-eluting stents (DES) = coated with medications that are slowly released to the site of the artery blockage in order to minimize excess tissue growth around the stent, thus reducing the risk of restenosis;<br>**6.** Bioabsorbable (bioresorbable) stents (BRS) = in time, these stents will be absorbed into the artery wall after it has healed, thus eliminating the need for medications that prevent the blood clots from forming in the stent and re-establishing a more physiological pattern of the coronary flow and shear stress. However, recent studies have shown that BRS have a high device thrombosis rate, thus being rarely used nowadays.
**3. Why is it done?**	**7.** PCI may be indicated for stable patients suffering from chest pain or shortness of breath, in order to reduce or eliminate these symptoms of stenosed coronary arteries (coronary heart disease). It might also be used to treat an acute coronary syndrome (heart attack, unstable angina) by quickly re-establishing blood flow through the affected vessel, thus decreasing the amount of damage to the heart and reducing mortality in these patients.
**4. What to do before the procedure**	The patient will need to prepare for the procedure by:<br>**8.** Informing the doctor about all the medicines and natural health products he/she takes, especially antiplatelet medicines (for example, aspirin, clopidogrel, prasugrel, ticagrelor), blood thinners (for example, acenocumarol, warfarin, dabigatran, edoxaban and rivaroxaban) or medicines for diabetes (for example, metformin). The doctor will tell the patient which medicines to take or stop before the procedure;<br>**9.** Informing the doctor about any present illnesses, contrast dye allergy and/or any potential pregnancy (in case of women with childbearing potential, as stenting implies use of X-rays);<br>**10.** Having several tests, such as blood tests, an ECG (electrocardiogram) and a coronary angiogram to localize the narrowed or blocked parts of the coronary arteries and assess the severity of the stenosis. Coronary angiography can be performed via invasive cardiac catheterization (case in which could be immediately followed by coronary stenting during the same procedure) or non-invasively via computed tomography coronary angiography. In certain cases, a stent may ultimately not be implanted;<br>**11.** Avoiding eating and drinking anything (except water), as well as smoking, for four to six hours before the procedure. Water can be consumed up to two hours before the procedure. Exact instructions will be given by the specialized staff;<br>**12.** Asking the doctor about anything that might be unclear. It is very important to completely understand every aspect of the procedure.
**5. The procedure**	**13.** Angioplasty normally takes between 30 minutes and two hours, but it might take longer.<br>**14.** The patient will be asked to remove any jewelry or other objects that might interfere with the procedure. They may wear their dentures or

	hearing aid if they use either of these. The patient will also remove his/her clothes and will be given a gown to wear.<br>**15.** It is important that the patient empties his/her bladder before the procedure.<br>**16.** The area of the catheter insertion (groin, arm, wrist) will be shaved, if necessary.<br>**17.** An intravenous line will be started in the hand or arm before the procedure, in order to allow the injection of medicines and the administration of IV fluids, if necessary.<br>**18.** The patient will be placed on his/her back on the procedure table and will be connected to an electrocardiogram (ECG) monitor that records the electrical activity of the heart using electrodes that stick to the patient's skin. Vital signs (heart rate, blood pressure, breathing rate, oxygen level) will be monitored during the procedure.<br>**19.** Angioplasty is performed using local anesthesia, meaning that the patient will be awake during the procedure, although light sedatives might be administered through the IV line to help the patient relax. A local anesthetic will be injected into the skin and soft tissues at the insertion site. This may cause a stinging sensation at the site for a few seconds.<br>**20.** Once the local anesthetic has taken effect, a small plastic tube called a sheath will be introduced into the chosen artery (groin, arm, wrist) with the help of a needle. Through the sheath, all the guidewires, catheters, stents and balloons will be advanced into the coronary arteries. Fluoroscopy will be used to help visualize the procedure. The patient might feel pressure in the area of insertion, but shouldn't feel sharp pain. Inform the doctor if you do so. Also, the patient will be unable to feel the catheters in his/her body.<br>**21.** Once the catheter is in place, contrast dye will be injected into the coronary arteries under X-ray control, in order to visualize the narrowed areas. During the procedure, the X-ray tube will move around the patient, in order to acquire multiple images of the coronary arteries from different angles. The injection of contrast dye might cause some effects such as a flushing sensation, salty or metallic taste in the mouth, or a brief headache, which last only for a few moments. Inform the doctor if you feel any breathing discomfort, sweating, numbing, itching, nausea, vomiting, chills or heart palpitations. Also, the patient may be asked to take in a deep breath and hold it for a few seconds while a series of rapid X-ray images will be taken.<br>**22.** After the narrowed area is located, if necessary, a thin wire and a tiny inflatable balloon will be advanced towards it. It will be inflated and deflated in order to dilate the stenosed segment, by pushing the plaque back against the artery wall. At this point, the patient might have chest pain or discomfort because of the temporary blockage of the blood flow due to the balloon inflation. Any chest pain or discomfort should go away after the balloon is deflated. Inform the doctor if you notice any continued discomfort or pain, such as chest pain, neck or jaw pain, back pain, arm pain, shortness of breath or other breathing problems.<br>**23.** Next, a stent will be inserted. It will be collapsed around another inflatable balloon. The stent will expand when the balloon is inflated and will remain in place when the balloon is deflated and removed, acting like a scaffold and holding the artery open. In some cases, more than one stent may be needed to open a blockage.<br>**24.** The procedure is considered finished once it has been determined that the artery is opened sufficiently. The wires and catheters will be removed, as well as the sheath, and the insertion site will be closed in one of various manners. It might be done by the use of sutures, with special closure devices, or by applying manual pressure over the area. Once the bleeding has stopped, a tight bandage will be placed on the site.<br>**25.** Staff will help the patient slide from the table onto a stretcher so that he/she can be taken to the recovery area.
**6. What happens after the procedure**	**26.** The patient will be taken to an observation ward, where the vital signs, as well as the insertion site, circulation, and sensation in the limb will be checked regularly. He/she will be asked to stay in bed, lying on his/her back, for a few hours. Eating and drinking will be permitted as soon as the patient feels like doing so. The patient will be encouraged to drink fluids in order to prevent dehydration and flush the dye from the kidneys. If the patient experiences any discomfort, pain medication may be given.<br>**27.** After the procedure, the doctor will prescribe medicine which is highly important to take as prescribed. These include aspirin (each day indefinitely, unless the patient is allergic to it or unable to tolerate it), anti-platelet drugs (clopidogrel, prasugrel, ticagrelor) and statins (to stabilize coronary atheromatous plaques), and may also include nitroglycerin (spray or tablet to put under the tongue in case of angina) if angina persists after stenting. Some of these medications will be required for at least one year after the procedure. Stopping the medication

	early greatly increases the risk of a heart attack by the treated artery becoming blocked.<br>**28.** The patient will be discharged when he/she is feeling well and has no angina. The length of the hospital stay depends on how quickly the patient is able to recover and perform some physical activity. If the procedure was needed during a heart attack, the hospital stay and recovery period will most likely be longer.
**7. What to know/to do at home**	**29.** It is possible to feel a slight discomfort in the chest after the procedure, but it is normal and it usually passes in a few days.<br>**30.** A bruise might appear on the skin where the catheter was inserted and it may be sore for a few days. If the patient experiences increased pain, swelling, redness, bleeding, other draining from the site (infection), fever or chills, it is important to contact the doctor.<br>**31.** Most people can start doing normal moderate-intensity activities as soon as they go home and can return to work within a week. Heavy lifting and straining should be avoided, as well as driving, for a week. It is highly recommended to attend a cardiac rehabilitation program.<br>**32.** Apart from taking the medication as prescribed, one should tend to the health of their heart by being smoke-free, maintaining a healthy weight, adopting a healthier diet, doing regular exercise and controlling other conditions, such as diabetes and high blood pressure. Angioplasty does not cure coronary artery disease and it does not reduce the risk factors. Thus, it is important to continue healthy lifestyle habits.<br>**33.** If any of the symptoms return, such as chest pain or shortness of breath, it is important to contact the doctor. If the patient experiences chest pain at rest or pain that doesn't respond to nitroglycerin, emergency medical help should be sought.<br>**34.** The stents are not affected by security systems at airports or MRI scans.
**8. Risks**	**35.** About one in five patients may suffer from common risks which include:<br>• bleeding or bruising at the site where the catheter was inserted (groin, wrist, arm);<br>• restenosis of the coronary artery (in less than 10% of cases if a DES was used).<br>**36.** Rare complications that might occur in up to 2% of cases include:<br>• kidney damage due to the contrast dye;<br>• allergic reactions to the contrast dye or other medication used;<br>• blood clots (thrombosis) inside the stent;<br>• heart attack;<br>• stroke;<br>• arrhythmias (irregular heartbeats);<br>• infection;<br>• excessive bleeding which would require blood transfusion;<br>• rupture or complete closing of the coronary artery; a stent can often fix this, but sometimes patients need emergency coronary artery bypass surgery;<br>• death.<br>**37.** The risk of developing complications is increased by the following factors:<br>• advanced age;<br>• poor general health;<br>• pre-existent kidney disease;<br>• multi-vessel disease (more than one coronary artery is blocked);<br>• history of serious heart disease (such as heart failure, previous heart attacks);<br>• whether the procedure was planned or performed as emergency treatment (the latter is always riskier because of the need for quick

	intervention and the patient's unwell status).
**9. Benefits**	**38.** The benefits of angioplasties are observed immediately in most patients (over 90-95%), meaning that blood flow through the artery returns to normal or near normal. Even if complete relief might not be achieved in all cases, patients' symptoms will most likely improve, allowing them to be more active and comfortable.<br>**39.** Depending on their status, some patients might be discharged the same day or the following day.<br>**40.** The procedure exposes the patient to a certain amount of radiation. However, studies have shown that the risk of developing cancers does not exceed the risk of other procedures.
**10. Costs**	**41.** The cost of an angioplasty depends not only on the number and type of stents used, but also on many other various factors, due to the complex nature of the procedure, thus being difficult to give an exact estimate in advance. The cost of an intervention may vary around Europe from 2000-4000 Euros (Poland, Romania) to approximately 8000-10000 Euros in western countries (e.g. Spain, Germany).<br>**42.** Prices of implantable devices vary in Europe from around 70-100 Euros per stent for BMS to approximately 200-300 Euros per stent for DES and about 1000 Euros per stent for bioabsorbable stents.
**11. Alternative treatment approaches**	**43.** Coronary artery by-pass grafting is a type of open-heart surgery in which segments of veins or arteries from the patient's legs, arms or chest are used to create a new channel through which blood can be redirected past the blocked part of the artery. This procedure is usually recommended when multiple coronary arteries have become narrowed or blocked, or if the anatomy of the coronary arteries is abnormal, but it is a highly invasive procedure needing general anesthesia and a much longer and difficult rehabilitation process, so it may not be suitable for everyone.<br>**44.** Atherectomy consists of removal of plaque at the site of the narrowing of the artery. It is a procedure similar to angioplasty, in which the plaque is broken up or cut away with the help of a catheter. It is an adjunctive but highly important operation, done rarely in order to prepare the vessel before stenting.<br>**45.** Medication, along with important lifestyle changes, might be an effective alternative for patients with relatively mild symptoms and stable status, by reducing or eliminating some of the factors that contribute to artery disease. Medical management of artery disease also includes controlling any other health conditions that may affect artery health, such as diabetes or hypertension.
**12. Coronary atherosclerosis and diet**	**46.** It has been demonstrated that elevated serum cholesterol levels are one of the main indicators of susceptibility to atherosclerotic coronary heart disease (ASHD), along with serum triglyceride levels and serum lipoprotein levels. Several studies have shown the beneficial effects which certain foods have on levels of serum fats. It is recommended to reduce certain foods in the diet, such as saturated fats (found mostly in meat, eggs, dairy products), processed products and large amounts of salt and sugar, and increase the intake of fruits, vegetables and whole-grains.<br>**47.** However, while lifestyle and dietary changes combined with medical treatment may keep atherosclerosis from getting worse, they cannot actually reverse the disease. Patients in need of PCI should not delay or refuse necessary medical interventions relying on unproven interventions.
**13. Coronary artery disease and alternative treatments**	**48.** It is extremely important that patients take health-related decisions based on reliable sources of data. The quality, efficiency and risks of most advertised unconventional treatments are not based on solid scientific evidence.<br>**49.** Before trying any type of alternative treatment for coronary artery disease (such as acupuncture, yoga, chelation therapy, herbs – hawthorne, yarrow, garlic), patients are strongly advised to check whether it is evidence based.<br>**50.** Patients should always consult the medical practitioner before starting any alternative treatment, considering that some of them might even interfere with the current medical treatment. Patients should always purchase medications only from trusted sources.
